# A Portable High-Resolution Snapshot Multispectral Imaging Device Leveraging Spatial and Spectral Features for Non-Invasive Corn Nitrogen Treatment Classification

**DOI:** 10.3390/s25051320

**Published:** 2025-02-21

**Authors:** Xuan Li, Zhongzhong Niu, Ana Gabriela Morales-Ona, Ziling Chen, Tianzhang Zhao, Daniel J. Quinn, Jian Jin

**Affiliations:** 1 School of Agricultural and Biological Engineering, Purdue University, West Lafayette, IN 47907, USA; li1606@purdue.edu (X.L.); niu38@purdue.edu (Z.N.); zlchen@mit.edu (Z.C.); zhao770@purdue.edu (T.Z.); 2 Department of Agronomy, Purdue University, West Lafayette, IN 47907, USA; aona@purdue.edu

**Keywords:** multispectral imaging, spatial–spectral combined phenotyping, machine learning, nitrogen treatment classification

## Abstract

Spectral imaging has been widely applied in plant phenotyping to assess corn leaf nitrogen status. Recent studies indicate that spatial variations within a single leaf’s multispectral image provide stronger signals for corn nitrogen estimation. However, current technologies for corn multispectral imaging cannot capture a large corn leaf segment with high-resolution and simple operation, limiting their efficiency and accuracy in nitrogen estimation. To address this gap, this study developed a proximal multispectral imaging device that can capture high-resolution snapshot multispectral images of a large segment of a single corn leaf. This device uses airflow to autonomously position and flatten the leaf to minimize the noise in images due to leaf curvature and simplify operation. Moreover, this device adopts a transmittance imaging regime by clamping the corn leaf between the camera and the lighting source to block the environmental lights and supply uniform lighting to capture high-resolution and high-precision leaf images within six seconds. A field assay was conducted to validate the effectiveness of the multispectral images captured by this device in assessing nitrogen status by classifying the nitrogen treatments applied to corn. Six nitrogen treatments were applied to 12 plots of corn fields, and 10 images were collected at each plot. By using the average vegetative index of the whole image, only one treatment was significantly different from the other five treatments, and no significant difference was observed among any other groups. However, by extracting the spatial and spectral features from the images and combining these features, the accuracy of nitrogen treatment classification improved compared to using the average index. In another analysis, by applying spatial–spectral analysis methods to the images, the nitrogen treatment classification accuracy has improved compared to using the average index. These results demonstrated the advantages of this high-resolution and high-throughput imaging device for distinguishing nitrogen treatments by facilitating spatial–spectral combined analysis for more precise classification.

## 1. Introduction

Global corn production is facing significant challenges including climate change, depletion of natural resources, and increasing farming costs [1,2,3]. Moreover, the efficient management of nitrogen (N) fertilization is essential for optimizing corn yield and minimizing environmental impacts [4,5,6]. Conventional methods for evaluating nitrogen levels in corn, such as chemical analysis and invasive approaches, often damage plant structures, require extensive labor, and delay results [7,8,9]. In contrast, advances in multispectral imaging (MSI) technology now provide a non-destructive and rapid solution for capturing detailed canopy data, significantly improving the precision and scalability of nitrogen monitoring in corn fields [10,11,12].

Specifically, proximal MSI devices enable capturing images at a close range which leads to high-spatial-resolution images without losing spectral resolution. These devices facilitate the analysis of subtle changes in plant physiological characteristics related to nutrient deficiencies [13,14,15]. For example, the soil plant analysis development (SPAD) meter is one type of proximal MSI device. Multiple studies have demonstrated the effectiveness of using a SPAD meter to estimate nitrogen status in corn leaves [16,17,18]. However, SPAD meters only measure a dot area, which compromises the result accuracy since the distribution of nutrients in a leaf is not uniform [19,20,21]. Therefore, proximal MSI devices that capture larger amounts of leaves are beneficial for more comprehensive analysis that leverages both spatial and spectral resolution.

Several studies have demonstrated the potential of leaf-level MSI devices in plant phenotyping. Li et al. (2023) [22] developed a soybean leaf multispectral imaging device that improved the imaging speed to less than five seconds, and the device was able to detect the effect of nitrogen treatment on field-grown soybeans. However, the devices developed in these studies all had a small field of view, which made them incompatible with imaging the long and wide corn leaves. In another study, Zhang et al. (2019) [23] developed a multispectral corn leaf scanner that captures corn leaf images by sliding along the leaf. The data collected using this device showed a strong correlation with SPAD meter measurement, inferring the potential of using this device to determine the nitrogen status in a corn leaf. However, the device developed in the study required the user to capture multiple images and stitch them together to reconstruct the whole-leaf image. This process introduced a noise source and made the device complicated to use.

These achievements have proved the effectiveness of leaf-level MSI devices in differentiating nitrogen status. However, the current devices cannot capture a large segment of a corn leaf in a snapshot manner, which has reduced the throughput and introduced additional noise during post-processing [24]. Furthermore, since a recent study has shown that corn nitrogen status estimation has improved when analyzing spatial–spectral features of leaf-level hyperspectral images instead of using an averaged spectrum [25], it is worth investigating if these enhancements can be replicated in multispectral imaging, thereby creating a more efficient and accurate method for assessing corn nitrogen status.

In this paper, we present a novel portable snapshot multispectral leaf-level imaging device for corn. This device uses airflow to reposition and flatten the corn leaf to improve morphological consistency and minimize the impact of corn leaf curvatures. Moreover, the device blocks environmental lights and uses a backlighting–transmittance imaging regime to capture high-resolution and accurate corn leaf images, in which the secondary veins are clearly visible. Such high resolution enables the analysis of spatial–spectral features and enhances the accuracy of classifying nitrogen treatments. In summary, this paper has established the following objectives:

1. Design and build a portable multispectral device that has a large field of view to capture high-resolution and accurate snapshot images of corn leaves.
2. Extract spatial–spectral features that have a better correlation with corn nitrogen treatment comparted to averaged spectral features.
3. Develop a machine-learning-based model that leverages both spatial and spectral data to better distinguish nitrogen treatment.

## 2. Hardware Development

This section explains the device hardware design, including the working principle and major part specifications and the operation flow for capturing images.

### 2.1. Overall Hardware Design

**Figure 1:**
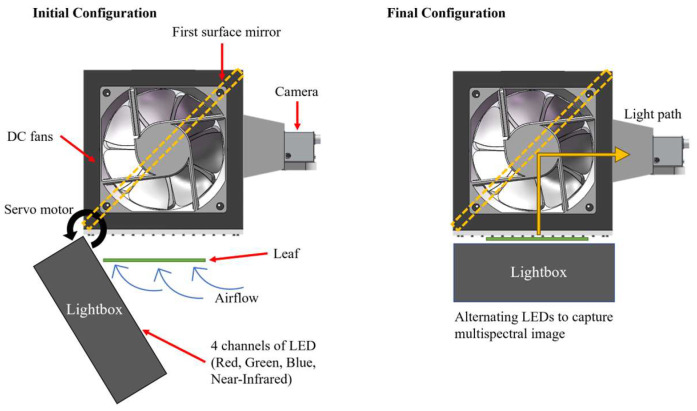
Working principle and schematic drawings of the device.

Figure 1 shows a schematic of the working principle of the snapshot multispectral imaging device, and its operation involves the following key components and principles:

(1) Leaf Positioning: This device uses high-power DC fans to create airflow to gently reposition and flatten the corn leaf within the imaging chamber. This process improves morphological consistency and minimizes distortions caused by natural leaf curvature, leading to clearer and more accurate images.
(2) Controlled Illumination: To reduce the impact of external lighting conditions, the device blocks environmental light by using non-transparent materials and utilizes a backlighting–transmittance imaging regime to create an enclosed imaging environment. This method illuminates the leaf from the bottom, enhancing the visibility of internal structures, such as secondary veins. Moreover, the lightbox closing mechanism was driven by a servo motor to achieve smooth and automatic operation.
(3) Multispectral Imaging: This device houses a high-resolution camera and a lightbox that includes narrowband light-emitting diodes (LEDs) with different wavelengths ranging from visible to near-infrared. This approach to creating multispectral images simplifies the design of the device compared to a spectral filter design or multiple-camera design. Additionally, this device uses a first surface mirror to alternate the light path. This design allows the device to achieve a larger field of view without increasing the device’s dimension, which improves portability and usability.
(4) Data Acquisition and Processing: The imaging process is rapid, capturing the necessary data within seconds to achieve high throughput. The captured images are processed in real time by an onboard microcontroller and saved to local storage. Additionally, the images were transmitted via Bluetooth to a smartphone application for immediate quality checking and editing.

### 2.2. Hardware Specifications

**Figure 2:**
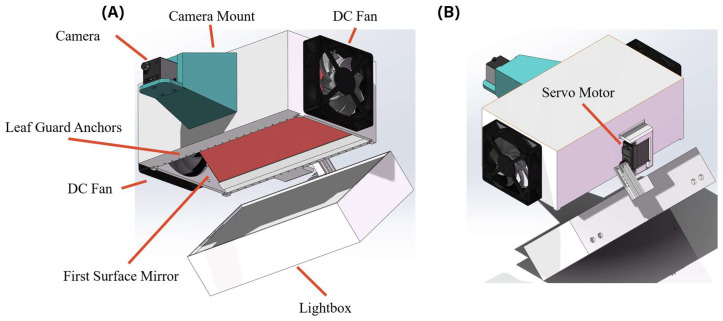
The 3D model of the device: (**A**) front view; (**B**) rear view.

Figure 2 shows the 3D model of the novel snapshot multispectral imaging device with major components and parts indicated. This device is made with 3D printed parts and off-shelf parts. The 3D printed parts included the main body, lightbox, and camera housing, and the off-shelf parts included the first surface mirror, DC fans, camera, LEDs, servo motor, and microcontrollers. There was also a handle attached to this device, but it is omitted in this figure for clarity.

The dimensions of the main body were 250 × 148 × 100 mm (L × W × H), and the dimensions of the lightbox were 250 × 128 × 55 mm (L × W × H). The camera used in this device was a monochrome camera produced by Teledyne FLIR LLC. The resolution of the camera was 1920 pixels × 1200 pixels (horizontal × vertical). A low-distortion (−1.0%) lens with an 8 mm fixed focal length manufactured by Kowa Optronics Co., Ltd. (Tokyo, Japan) is attached to the camera as an object lens. With these components, the resulting horizontal field of view (HFOV) was 69.67°, and the vertical FOV (VFOV) was 47.45°.

To capture a large segment of corn, the working distance needs to be increased. A longer working distance was achieved by using a first surface mirror and adding a camera mount. The first surface mirror was made by order from First Surface Mirror LLC. The mirror thickness was 3 mm, and it was made with glass. The reflectivity was greater than 94% for visible light regions and greater than 85% for near-infrared regions based on the manufacturer-provided specifications. By adding a camera mount to further increase the working distance, the resulting image covers an area with a size of 240 × 110 mm (horizontal × vertical) which includes 2/3 of a V6 corn leaf.

On the main body, linearly distributed holes were added to serve as the anchors of the leaf guard. The leaf guard was a grid made with five 0.2 mm thick nylon threads anchored to those holes. These ultra-fine monofilament nylon threads were stiff and strong enough to flatten the leaf and hold the leaf at a fixed position to achieve a consistent imaging distance. Moreover, only five threads were used to form the leaf guard to minimize the covered area on the corn leaf; their impact on the image could be neglected.

The DC fans used to create the airflow were manufactured by Mechatronics Fan Group; each of the fans had dimensions of 92 × 92 × 38 mm (L × W × H) and had a nominal 4800 rotation per minute (RPM) max under 12 V input. These fans created negligible vibrations and weighed only 180 g, making them suitable for portable applications.

The servo motor used to drive the lightbox was MG995, manufactured by TowerPro (Carson City, NV, USA). The servo motor weighed 55 g and had a stall torque of 11 kg/cm under 6 V input, making it ideal for operating the lightbox while minimizing the weight of the device.

The lightbox of this device was the most critical component for capturing clear and accurate images. This device imaged a single leaf to obtain the transmittance multispectral images of four colors, including blue, green, red, and near-infrared (NIR), and the peak wavelength for each color was 460 nm, 525 nm, 630 nm, and 850 nm, respectively. The LED strips were manufactured by Waveform Lighting, and the following table, Table 1, describes the detailed specifications and cost provided by the manufacturer for each color used in this device.

The lightbox housed a total of 29 LED strips (12 LEDs/strip): five LED strips for blue, green, and red colors and 14 LED strips for NIR. Because the NIR LEDs had low intensity, the number of LED strips was significantly increased compared to visible colors. The lightbox was covered by a 2 mm thick Teflon panel and a 2 mm thick LED diffusion sheet to improve the light uniformity.

The device was operated using a 12 V rechargeable portable battery as its energy source. This battery was converted to 5 V to supply power to the Raspberry Pi 4B, which functioned as the device’s controller. The lightbox and DC fans were also powered by the same battery, but a 12 V-12 V voltage stabilizer was used to keep the LEDs’ brightness consistent.

**Figure 3:**
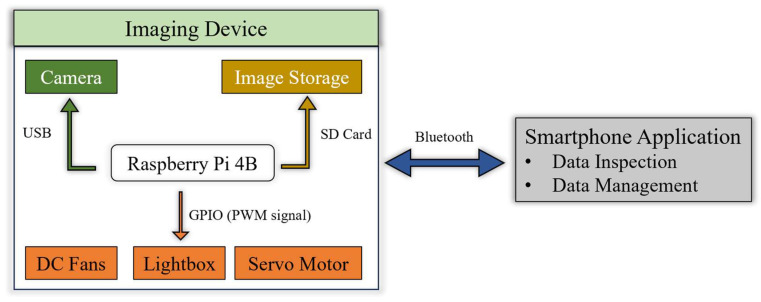
Data flow and communication protocol of the device.

The Raspberry Pi 4B, programmed in Python 3.7.11, generated pulse-width modulation (PWM) signals to activate the LEDs, turning DC fans on and off and commanding the servo motor through general-purpose input–output (GPIO) ports. Additionally, the microprocessor powered the imaging camera and managed the timing of image capture via its built-in USB 3.1 port, and the captured images were stored on a local SD card in Tagged Image File Format (TIFF). This device was designed to be used with a smartphone application, and communication was established via Bluetooth. Figure 3 shows the data flow and the communication protocol between each major component of this device.

### 2.3. Operation Flow for Non-Invasive Imaging

**Figure 4:**
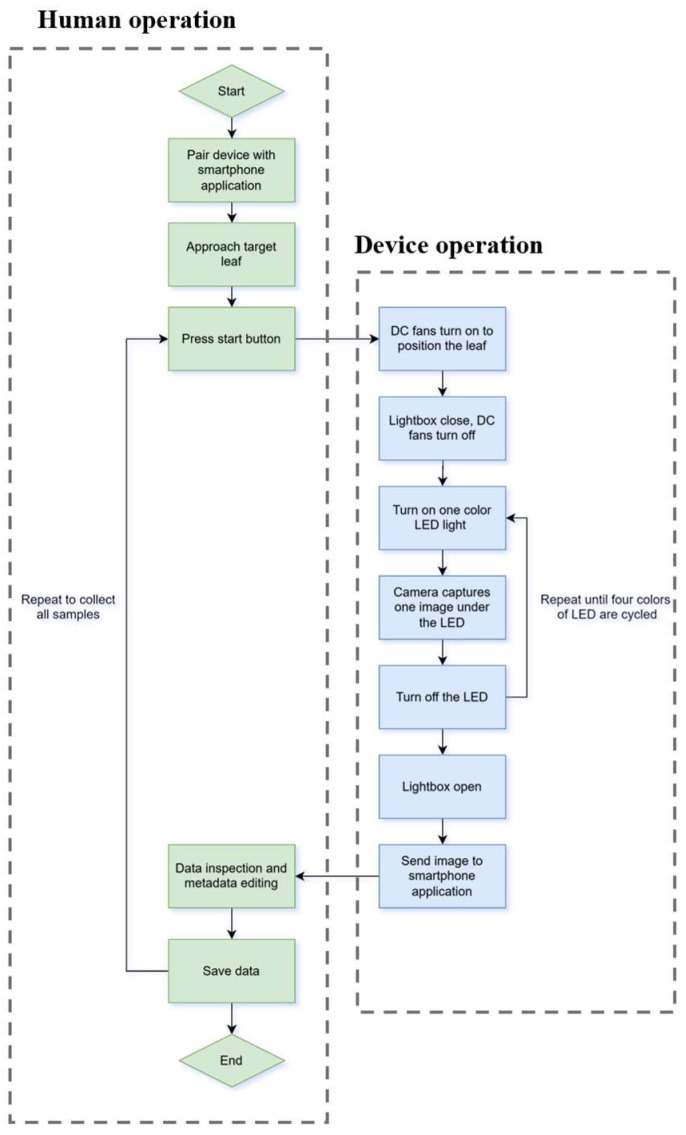
Operation flowchart.

Figure 4 shows the operational flow chart of this device. As previously discussed, this device was designed to be paired with a smartphone application for data inspection and management. Therefore, the first step was to pair the device with the smartphone application developed in a previous study [13].

After the device is paired with the smartphone, the user can approach the target leaf from the side. Once the device is put directly above the leaf and the vertical distance is less than 1 cm between the imaging chamber and the leaf, the user should press a physical button to initiate the imaging sequence.

After the button is pressed, two DC fans are energized to attract and hold the leaf. Then, the lightbox closes to block environmental lighting and flattens the leaf. The DC fans stop one second later after the lightbox is closed to make sure the leaf position is secured. When the DC fans stop, the first LED color turns on, and the camera captures one image. By repeating this process three times, four colors of LEDs are all cycled, and a multispectral image with four channels is captured. During this process, the user should hold the device as still as possible to prevent blurring in the final image. With an average imaging time of 5.2 s, a preview of the final image is transmitted to the smartphone application for the user to check the quality. If the quality is acceptable, the user can edit the image’s filename to record the metadata such as plant number or treatment of the subjects.

Compared to a previously designed multispectral imaging device [23], the operation procedure is significantly simplified, as the newly developed device captures snapshot images rather than spatial-scanning images. Moreover, the image quality has been improved as the impact of uneven scanning speed when using the scanning imaging device has been eliminated.

## 3. Image Processing

This section explains the pre-processing steps of the multispectral images captured by the device. The goal of the pre-processing is to improve the image quality by removing noise caused by uneven light distribution, background removal for leaf isolation and image reconstruction to remove the nylon thread.

### 3.1. Image Calibration

The first step of image processing was image calibration through white references. The goal of this step was to reduce the impact of unevenly distributed light [26,27]. The following equation was used to create calibrated images:

(1) $${Image}_{cal}=\frac{{Image}_{raw}}{{Image}_{white}}$$

In this equation, the uncalibrated original image is represented by Image_raw_. The reference image of a white-colored plastic board is taken under each color of light, and it is represented by Image_white_. By imaging a white-colored board, the light distribution can be captured and used for compensating the noise induced by nonuniformly distributed light. Since there are four colors of light in each multispectral image, each color has its own Image_white_, and each color is calibrated individually. The Image_white_ for each color is captured before the data collection and it is used for all samples, because the nonuniformity of the light is purely caused by the LED distribution. The resulting image after this operation Image_cal_ represents the calibrated image that is used for the following processes.

### 3.2. Image Reconstruction

After the image was calibrated, the next step of the image processing was to remove the nylon threads. This study followed a method described in the work by Li et al. (2023) [22]. The first step was to identify the nylon threads using a 2-D Gabor filter because the nylon threads had distinct orientations compared to the pattern on the corn leaves. After the nylon threads were removed from the image, the inpainting function available in MATLAB 2024a was used to fill in the missing pixels.

### 3.3. Image Segmentation

The Normalized Difference Vegetation Index (NDVI) grayscale images were generated using the red (R) and near-infrared (NIR) bands. Based on the United States Geological Survey, the equation for calculating NDVI is shown below:

(2) $$NDVI=\frac{Image_{NIR}-Image_{R}}{Image_{NIR}+Image_{R}}$$

The utilization of the NDVI as a vegetation index effectively separates vegetation from the background [28,29]. Subsequently, the Segment Anything Method (SAM) [30] was employed for image segmentation. The entire image was used as the Region of Interest (ROI) for the SAM, which stabilizes the segmentation process, facilitating accurate differentiation of both healthy and stressed plants. These steps were executed in MATLAB 2024a, employing the Image Processing and Deep Learning Toolboxes.

**Figure 5:**
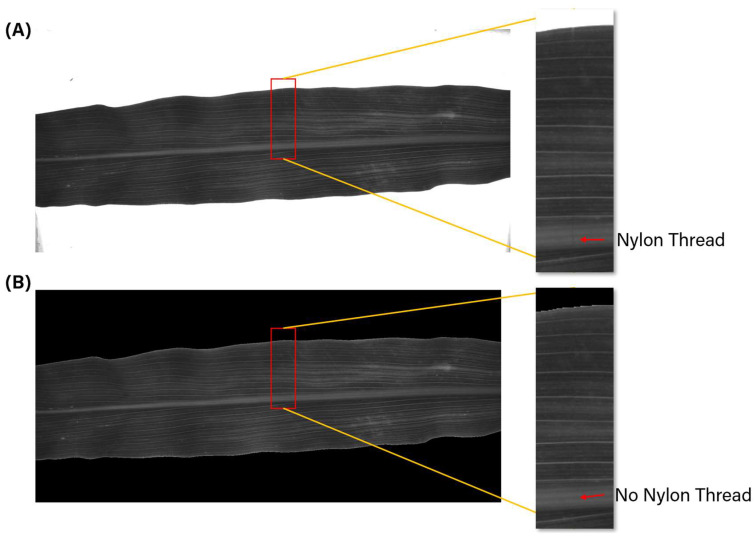
Example of processed image under red lights. (**A**) Before processing. (**B**) After processing.

Figure 5A,B show an example image before and after these processing steps, respectively. By comparing these figures, it can be observed that the bright background in Figure 5A was successfully removed. Moreover, the zoomed-in image on the right side of Figure 5A,B proves that the nylon thread was removed, and the underlying image details are restored.

### 3.4. Feature Extraction and Modeling

**Figure 6:**
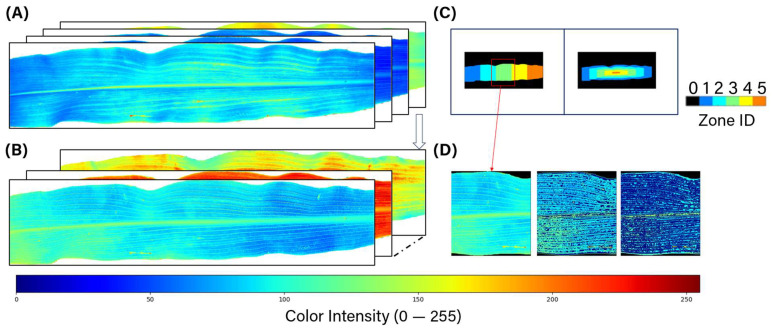
(**A**) Original multispectral images after segmentation with colormap. (**B**) Generated index heatmaps with colormap. (**C**) Corn leaf zoning method based on leaf shape. (**D**) Vein segmentation based on contour detection.

To fully utilize the spectral and spatial features of high-resolution corn leaf images, the multispectral images were processed, and the results are shown in Figure 6A. Six index heatmaps were generated by combining every two channels of the original bands using Equation (3), where $I_{c}$ represents a pixel value in the generated heatmap while $I_{a}$ and $I_{b}$ correspond to pixel values at the same position of two channels.

(3) $$I_{c}=\frac{{I_{a}-I}_{b}}{I_{a}+I_{b}}$$

To further utilize the spatial dimension, the veins were segmented using an edge detection method. Additionally, the leaf was subdivided into smaller sections based on their morphological features. For each section, various statistical measurements, including skewness [31], kurtosis, and the Gray-Level Co-occurrence Matrix (GLCM) [32], were calculated. These features help to quantify the smoothness, asymmetry, and sharpness of the leaf’s color.

With a substantial number of features extracted from MSI, a random forest model was employed to classify the nitrogen levels in the corn leaves. This technique is particularly effective for handling large datasets as it can manage thousands of input variables without necessitating variable deletion and is minimally affected by the curse of dimensionality [33]. Moreover, a random forest is adept at modeling complex, non-linear relationships that are beyond the scope of linear models [34]. This method demonstrates the efficacy of integrating spectral–spatial analysis to enhance traditional metrics, such as the average color index and NDVI. To validate the model’s reliability, a 10-fold cross-validation was implemented.

## 4. Field Experiment for Nitrogen Treatment Classification

This section discusses field experiment design and the nitrogen treatment classification results using the newly developed multispectral imaging device.

### 4.1. Plant Materials

**Figure 7:**
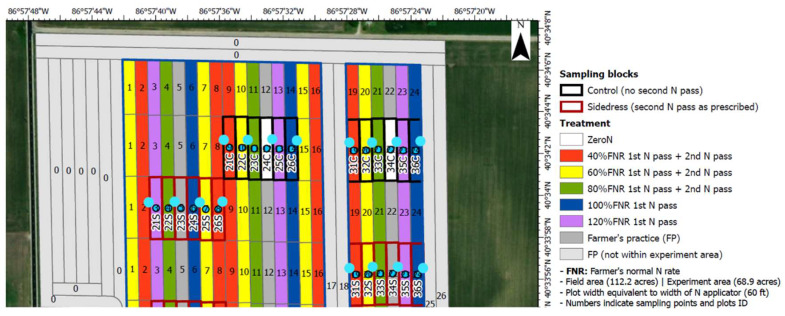
Field map.

The corn plants used in this study were grown in collaboration with a local farmer in 2023 in White County, Indiana, USA. Figure 5 shows the field’s location and the corresponding treatment for each plot. In this study, the plots without a second N pass (control blocks which are enclosed by black rectangles in Figure 7) were used for device validation. Specifically, plots 21C–26C and 31C–36C were used for data collection. There were six types of nitrogen treatments applied to this field. For each treatment, it was identified as a percentage of a farmer’s normal N rate. For example, 40% means that the amount of nitrogen applied to that plot was 40% of the farmer’s normal N rate. In each plot, 10 corn plants were randomly selected, and the top collared leaf of each plant was imaged at stage V6, for a total of 120 images.

### 4.2. Results and Discussion

#### 4.2.1. Nitrogen Treatment Identification Using Whole-Leaf Averaged Index

NDVI is used as the reference for nitrogen study and serves as a reliable indicator of plant health by leveraging differences in light reflectance at specific wavelengths to assess chlorophyll content and biomass [35,36,37,38]. Numerous studies have demonstrated its effectiveness in monitoring nitrogen status and overall crop vitality, making it a fundamental tool in precision agriculture and high-throughput phenotyping [17,38]. Therefore, by using Equation (2), the NDVI value for each pixel on the leaf was calculated. Then, these calculated values were averaged to obtain a single number to represent the whole leaf. The single value was used to identify nitrogen treatment.

**Figure 8:**
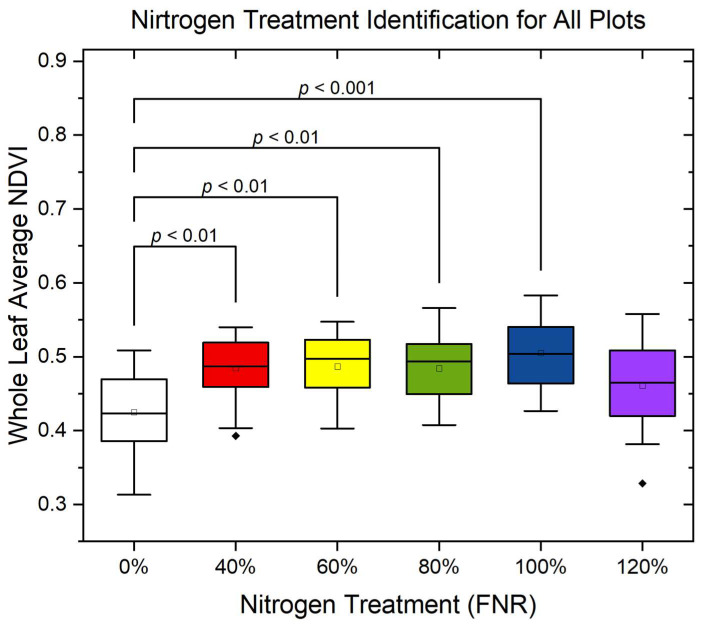
N treatment identification results based on whole-leaf averaged index.

Figure 8 shows the N treatment identification results for plots 21C–26C and 31C–36C. The *p*-values plotted on the figures indicate the significant statistical difference between each group. The *p*-values for groups that are not significantly different are not plotted in this figure. The *p*-values suggested that only the 0% N treatment can be clearly identified from the other treatments, and the other treatment groups are not significantly different from each other. Though the differences are expected, the less pronounced difference in NDVI among each treatment suggests that additional factors, such as environmental conditions or soil properties, may influence nitrogen uptake. Moreover, the whole-leaf average NDVI value drops under the 120% FNR treatment, which potentially indicates the plants develop stress symptoms under excessive nitrogen.

#### 4.2.2. Nitrogen Treatment Identification Using Extracted Spatial–Spectral Combined Feature

**Figure 9:**
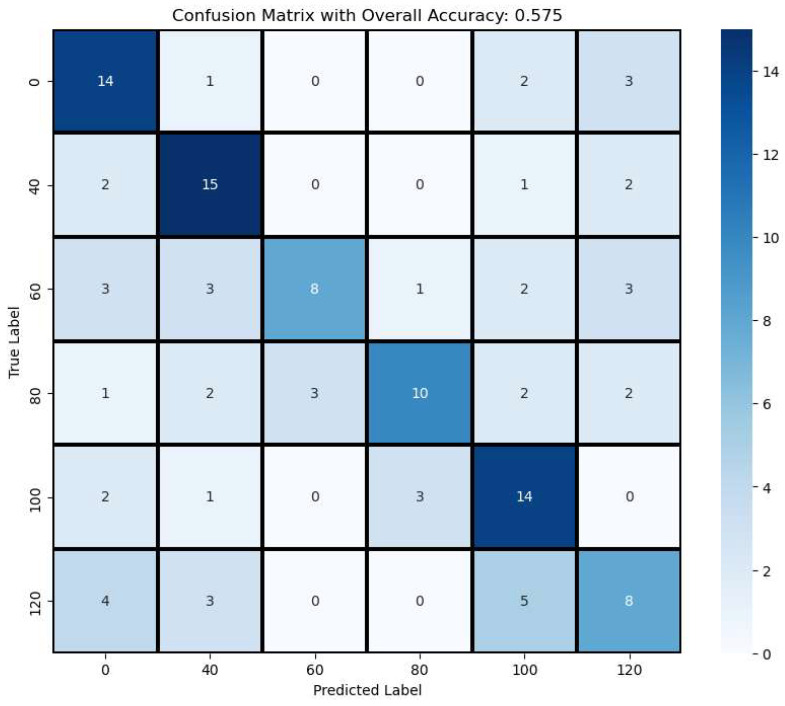
Identification results of N treatment using extracted spatial–spectral features.

The confusion matrix, shown in Figure 9, of the random forest model, built using spatial–spectral features extracted from MSI, achieved an overall accuracy of 0.575. Notably, treatment groups with 0%, 40%, and 100% nitrogen application rates displayed high classification accuracy, demonstrating the model’s capability to discern varying nitrogen levels based on MSI data. However, the 120% FNR group’s classification results aligned with trends observed in average NDVI; with over-fertilization, the nitrogen signal appeared weaker than in the 100% FNR group, posing a challenge for the model to distinctly categorize this group. Further research could investigate these findings, suggesting the possibility that excessive fertilizer use may have detrimental effects on corn plants.

Similarly, the classification results of the middle-level treatment (60% and 80%) also demonstrated lower accuracy. These misclassifications mostly occur between lower nitrogen treatments, which could suggest that similar corn growth can be achieved with lower nitrogen application, inferring the potential of reducing nitrogen fertilizer usage without impacting corn growth. Since corn growth is strongly correlated with the actual nitrogen content within the plant, which is influenced by environmental factors such as rainfall, temperature, and soil type, current nitrogen treatment classification approaches may not fully capture true nitrogen uptake [39,40]. In future studies, a more comprehensive model integrating multiple sensors—including this novel multispectral sensor—along with environmental and biochemical data, will be obtained to improve nitrogen content estimation and enhance classification accuracy.

Lastly, by using the average NDVI, only the 0%-nitrogen-treated corn plants were distinguished from other treatments, but treatments such as 40% FNR did not show significant differences when compared to other treatments, based on the t-test results. This suggests that incorporating additional features could significantly enhance the accuracy of the analysis and reveal meaningful information concealed within the leaf structures. This spatial–spectral analysis on MSI highlights not only the successes but also the opportunities to refine and optimize the model. Future efforts will involve developing and incorporating additional feature extraction methods aimed at improving model performance and advancing our predictive capabilities in agricultural applications.

## 5. Conclusions

This study developed a portable, high-resolution snapshot multispectral imaging device for non-invasive nitrogen treatment classification in corn. By integrating airflow-assisted leaf positioning and a backlighting–transmittance imaging regime, the device improves image clarity and morphological consistency, enabling more accurate nitrogen status estimation based on treatment classification. Field validation demonstrated that the spatial–spectral features extracted from multispectral images enhanced nitrogen treatment differentiation compared to traditional whole-leaf averaged NDVI. The random forest model successfully classified multiple nitrogen levels, particularly distinguishing 0%, 40%, and 100% FNR. Overall, this study highlights the advantages of snapshot multispectral imaging in improving nitrogen treatment classification accuracy and contributes to advancing precision agriculture technologies for efficient nitrogen management in corn production.

## Figures and Tables

**Table 1:** LED specifications and cost.

Color	Red	Green	Blue	NIR
Wavelength (Peak)	630 nm	525 nm	460 nm	850 nm
Wavelength (Dominant)	623 nm	530 nm	465 nm	N/A
Full width at half maximum (FWHM)	15.5 nm	30 nm	19.5 nm	10 nm
Cost	0.16 USD/LED	0.16 USD/LED	0.16 USD/LED	0.06 USD/LED

## Data Availability

The data is confidential.
